# Editorial: Exploring the multidimensional impact of homelessness on health and social inclusion

**DOI:** 10.3389/fpubh.2025.1749189

**Published:** 2025-12-18

**Authors:** Monica Amadini, Evasio Pasini, Francesco Puccio

**Affiliations:** 1 Research Centre on Family and Childhood Education, Department of Pedagogy/Education, Catholic University of Sacred Heart, Brescia, Italy; 2 Department of Clinical and Experimental Sciences, University of Brescia, Brescia, Italy; 3 Institute of Human Health, Ruaha Catholic University, Iringa, Tanzania; 4 Volunteer Association ‘Un Medico x Te', Brescia, Italy

**Keywords:** homelessness, health, social inclusion, caring approaches, multidisciplinary training

## Introduction

Homelessness constitutes a global crisis extending far beyond the mere absence of adequate shelter. It is a multifaceted phenomenon marked by severe housing deprivation, profound social marginalization, and systematic exclusion from fundamental human rights, including access to healthcare (1). As an expression of extreme social inequality, homelessness incurs devastating consequences: educational deficits, reduced life expectancy, increased morbidity and mortality, alongside significant societal costs such as elevated healthcare expenditure, diminished public safety, and higher criminal justice expenses.

The scholarly investigation of homelessness has evolved substantially over recent decades, drawing on diverse disciplines, including public health, sociology, psychology, medicine, social work, and urban studies. This interdisciplinary stance recognizes that homelessness cannot be satisfactorily understood or addressed through a single-discipline lens. Instead, it requires integrated approaches acknowledging the complex interplay between individual vulnerabilities, interpersonal relationships, community resources, systemic barriers, and structural inequalities.

Contemporary comprehension is firmly grounded in the social determinants of health framework, articulated by the World Health Organization (2) and numerous scholars (3, 4). This framework emphasizes that health is not solely a biological or medical concept but is fundamentally shaped by “the circumstances in which people are born, grow, work, live, and age, and the wider set of forces and systems shaping the conditions of daily life” (WHO, 2008). Health encompasses physical, mental, emotional, social, and spiritual dimensions, all profoundly compromised by homelessness. The framework distinguishes structural determinants—including socioeconomic position, social class, gender, and ethnicity—from intermediary determinants such as housing, working conditions, social support, and nutrition. Homelessness represents an extreme convergence of adverse determinants that produce severe health inequities.

Despite increasing attention to homelessness across multiple disciplines and the deployment of various national and international welfare policies, its full impact—both socially and from a healthcare perspective—remains insufficiently analyzed.

For instance, significant knowledge gaps persist concerning how homelessness intersects with social issues such as gender-based violence, substance misuse, mental health disorders, and traumatic brain injury.

## Aims and objectives

In alignment with the UN General Assembly's 2023 report titled “Inclusive policies and programmes to address homelessness”, the aim of our Research Topic was to explore homelessness' multidimensional nature and intersectionality with other social challenges, thereby advancing understanding and contributing to more effective solutions.

The specific objectives comprised:

1. Gathering empirical evidence on health and social living conditions of people experiencing homelessness from varied geographic contexts;
2. Examining intersections with concurrent challenges including mental illness, substance use, traumatic brain injury, and gender-based vulnerabilities;
3. Critically evaluating existing policies, programmes, and interventions addressing homelessness;
4. Showcasing innovative, holistic approaches integrating multiple forms of support;
5. Providing evidence to inform more effective, culturally responsive, rights-based global responses to homelessness.

Contributions employing quantitative, qualitative, and mixed-methods approaches were solicited, welcoming perspectives from multiple disciplines and regions. The resultant Research Topic of seventeen articles fulfills these ambitions, collectively emphasizing the need for holistic, intersectional, structurally informed responses to homelessness.

## Summary of contributions

The contributions span five continents and showcase remarkable disciplinary breadth, collectively illuminating homelessness' multidimensional nature while providing actionable insights for policy and practice.

## Healthcare system responses and service utilization

Liu et al., provide a comprehensive review of hospital and healthcare system responses to homelessness. They propose a practical framework integrating homelessness services within hospital settings that includes screening, care coordination, discharge planning, and community partnerships. This framework exemplifies a “health in all policies” approach recognizing housing as a fundamental determinant of health.

Karpenko et al., using retrospective secondary data from Berlin, reveal that people experiencing homelessness have significantly longer hospital stays compared with housed patients, controlling for medical complexity. This reflects challenges in discharging patients to appropriate post-acute care facilities due to lack of stable housing, underscoring how social determinants profoundly influence healthcare utilization and outcomes.

Graf et al., report findings from a German nationwide cross-sectional seroprevalence study examining immunity to measles, mumps, rubella, and varicella among homeless individuals. Large gaps in vaccine-mediated immunity were found, indicating substantial vulnerability and public health implications linked to housing instability.

## Intersectional vulnerabilities and concurrent disorders

Warren et al.'s qualitative study reports the barriers and facilitators that affect access to housing and health services for homeless with acquired brain injuries by analyzing data from the British Columbia Consensus for Brain Injury, Mental Health and Addiction project. The findings highlight the complexity of needs that siloed services cannot adequately address, advocating for integrated, trauma-informed care models.

Holliday et al., reveals that Native Hawaiian or Pacific Islander (NHPI) homeless veterans are 82% more likely to have a traumatic brain injury (TBI) diagnosis compared to non-NHPI homeless veterans analyzing of electronic medical records from the Department of Veterans Affairs (VA) spanning 2005–2024. However, NHPI homeless veterans were found to be significantly more likely to access and utilize VA services across various settings with positive results. This increased engagement underscores the urgent need for culturally responsive interventions tailored to Indigenous veterans' unique experiences.

## Programme evaluation and policy analysis

Wilkinson et al., examine over 600,000 veteran families served by the Supportive Services for Veteran Families Programme in the United States. Their descriptive analysis documents a distinctive model for combating homelessness: this programme provides essential assistance not only to homeless veterans themselves but also to their families, recognizing that homelessness is an issue with potential intergenerational consequences that requires a holistic, family-centered approach.

Young et al., use geospatial methods to evaluate San Francisco's homeless encampment clearance policies, revealing patterns in encampment locations and enforcement activities. Their findings raise ethical and efficacy concerns about punitive approaches, proposing spatial methodologies as valuable tools for policy evaluation.

Ogbonna et al., offer a systematic review of COVID-19 interventions targeting people experiencing homelessness across multiple countries. While innovative strategies—such as isolation facilities, improved hygiene measures, and vaccination programmes—were employed, persistent gaps in protection highlight the insufficiency of emergency responses compared to comprehensive long-term solutions addressing homelessness' root causes.

## Community-based and participatory approaches

Pasini et al., present an ethnographic case study of the volunteer association “A Doctor for You” in Brescia, Italy, revealing how grassroots organizations complement formal healthcare by delivering flexible, culturally sensitive care grounded in respect for human dignity.

Van Everdingen et al., explore community relations among homeless populations in the Netherlands. The results of this study show that despite the presence of assistance and welfare facilities in the area, the homeless population feel abandoned, revealing that the interaction between the homeless and the current system is a failure. They demonstrate how social connections, mutual support, and community belonging foster resilience despite ongoing housing instability, challenging deficit-based narratives and highlighting the agency and social capital of people experiencing homelessness.

Yohannes et al., utilize a photovoice methodology to document the experiences of homeless mothers in Addis Ababa, Ethiopia. Their research foregrounds the gendered dimensions of homelessness in low-income contexts, emphasizing the exacerbating impact of absent social safety nets and extreme poverty on women and children, while exemplifying the power of participatory research centered on lived experience.

## Health status, outcomes, and disparities

Bedmar et al., combine quantitative health assessments with qualitative interviews to assess the health status and self-perception of health among homeless individuals in Spain. They identify high prevalence of chronic and infectious diseases alongside complex interactions between objective health status and subjective perceptions, underlining the multidimensional nature of health in this population.

Richard et al., conduct a matched cohort study in Toronto, Canada, analyzing all-cause mortality disparities among homeless populations during the COVID-19 pandemic. Their findings demonstrate dramatically elevated mortality rates compared to housed populations, with exacerbated disparities during the pandemic, highlighting urgent needs for protective and structural interventions.

Mangrio's qualitative interviews with health, social care, and civil society professionals in southern Sweden illuminate concerns regarding young people in unstable housing situations. The study emphasizes barriers to access and the necessity for youth-targeted preventive approaches to disrupt pathways into chronic homelessness.

## Broader social context and determinants

Additional studies enrich the understanding of homelessness within wider social deprivation and inequality contexts. For example, research on COVID-19 mortality related to social deprivation in Mexico (Martinez et al.), the interplay between educational attainment, lifestyle, and health in China (Xiong et al.), and Chinese citizens' satisfaction with social security systems (Guo et al.) elucidate how homelessness intersects with broader patterns of social stratification, resource distribution, and structural violence.

These perspectives underscore that homelessness cannot be isolated from systemic factors such as education, employment, social protection, and societal attitudes which shape risks and responses.

## Impacts

Collectively, the contributions advance understanding on several fronts.

The profound health consequences across physical and mental illnesses, traumatic injuries, and premature mortality position homelessness as fundamentally a health crisis. The added costs and complications in healthcare utilization reinforce the need for upstream investment in housing and social supports to improve outcomes and reduce system burdens.

Moreover, the intersectional lens reveals how homelessness intersects with multiple vulnerabilities and marginalisations—mental health, substance use, traumatic brain injury, gender-based violence, and ethnicity—necessitating integrated, holistic care beyond siloed service models.

In this direction the evaluative work of policies and programmes highlights both promising innovations and persistent limitations in homelessness responses. Critiques of fragmented services and punitive enforcement contrast with evidence supporting Housing First, integrated care, community initiatives, and participatory methods.

These results advocate, advancing more effective interventions worldwide, such as: investing in affordable housing and subsidies; developing integrated service models addressing complex needs; adopting trauma-informed, culturally sensitive approaches; implementing upstream prevention tackling root causes; centring people with lived experience in designing and leading responses.

## Conclusion

Homelessness epitomizes a critical social challenge underscored by profound inequities that violate fundamental rights and yield severe consequences for individuals and communities. The multidisciplinary studies assembled here affirm that homelessness can be addressed through holistic strategies acknowledging its complexity.

The social determinants of health framework offers a unifying conceptual foundation, emphasizing the necessity of multi-sectoral and multi-level action—from individual support to systemic reform—to effectively eradicate homelessness.

The editorial team expresses gratitude to all contributors, reviewers, and the Frontiers editorial staff for their collective effort. Most importantly, recognition is extended to people experiencing homelessness whose lived realities inspire and ground this work. Commitment remains to ensure research serves their interests and advances their rights to housing, health, and dignity.
